# Supplementary material for: Transfer Learning in Magnetic Resonance Brain Imaging: A Systematic Review
Source: J Imaging. 2021 Apr 1;7(4):66. doi: 10.3390/jimaging7040066 (PMC8321322; doi:10.3390/jimaging7040066)
Supplement: Supplementary file 1 [file jimaging-07-00066-s001.zip › SupplementaryMaterial_FigureS1.pdf]

Supplementary Materials: Transfer Learning in Magnetic Resonance Brain Imaging: a Systematic Review

Juan Miguel Valverde [1], Vandad Imani[1,†], Ali Abdollahzadeh[1,†], Riccardo De Feo[1,†], Mithilesh Prakash[1,†], Robert Ciszek[1,†] and Jussi Tohka [1,*]

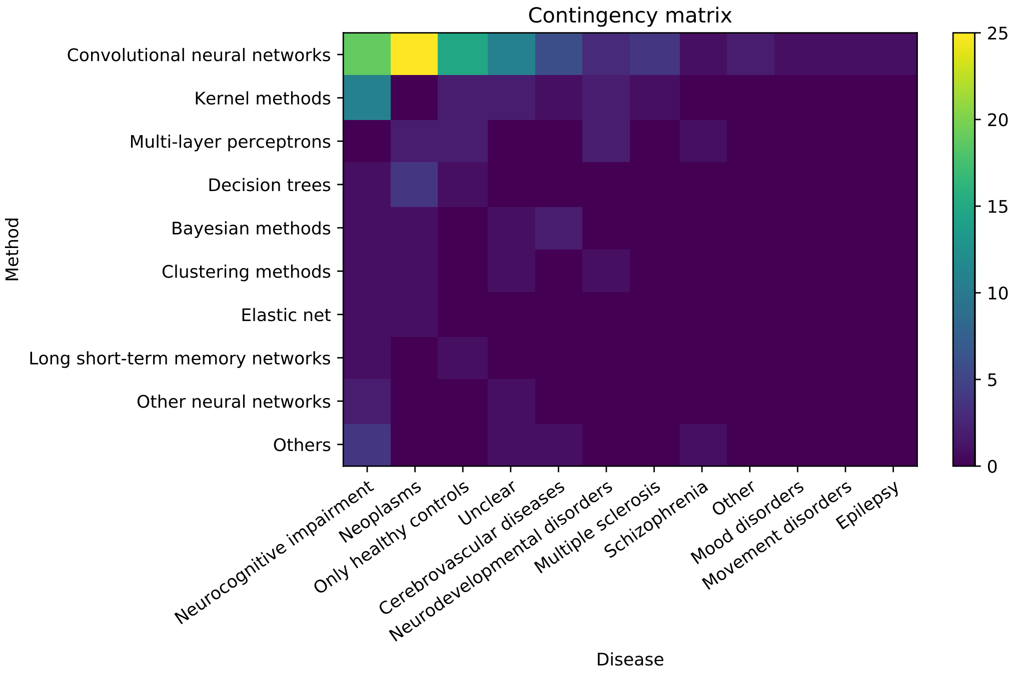

Figure S1. Contingency matrix between the machine learning methods and the brain disease categories where the methods have been applied to. Each element (i, j) of the matrix indicates the number of articles that applied machine learning method i to answer a question related to the brain disease j (or healthy controls).
